# Clinical outcomes of linezolid and vancomycin in patients with nosocomial pneumonia caused by methicillin-resistant *Staphylococcus aureus* stratified by baseline renal function: a retrospective, cohort analysis

**DOI:** 10.1186/s12882-017-0581-y

**Published:** 2017-05-22

**Authors:** Ping Liu, Blair Capitano, Amy Stein, Ali A. El-Solh

**Affiliations:** 1 Clinical Pharmacology, Global Established Pharma Business, Pfizer Inc, Groton, CT USA; 2 Medical Affairs, Specialty Care, Pfizer Inc., Collegeville, PA USA; 3 Customer Solutions, Biostatistics, Quintiles, Durham, NC USA; 4 Division of Pulmonary, Critical Care and Sleep Medicine, University at Buffalo, Buffalo, NY USA; 5 VA Western New York Healthcare System, Medical Research, Bldg. 20 (151) VISN02, 3495 Bailey Avenue, Buffalo, NY 14215-1199 USA

**Keywords:** Outcomes, Linezolid, Vancomycin, Pneumonia, MRSA, Renal function

## Abstract

**Background:**

The primary objective of this study is to assess whether baseline renal function impacts treatment outcomes of linezolid and vancomycin (with a dose-optimized regimen) for methicillin-resistant *Staphylococcus aureus* (MRSA) pneumonia.

**Methods:**

We conducted a retrospective cohort analysis of data generated from a prospective, randomized, controlled clinical trial (NCT 00084266). The analysis included 405 patients with culture-proven MRSA pneumonia. Baseline renal function was stratified based on creatinine clearance.

Clinical and microbiological success rates and presence of nephrotoxicity were assessed at the end of treatment (EOT) and end of study (EOS). Multivariate logistic regression analyses of baseline patient characteristics, including treatment, were performed to identify independent predictors of efficacy. Vancomycin concentrations were analyzed using a nonlinear mixed-effects modeling approach. The relationships between vancomycin exposures, pharmacokinetic-pharmacodynamic index (trough concentration, area under the curve over a 24-h interval [AUC_0–24_], and AUC_0–24_/MIC) and efficacy/nephrotoxicity were assessed in MRSA pneumonia patients using univariate logistic regression or Cox proportional hazards regression analysis approach.

**Results:**

After controlling for use of vasoactive agents, choice of antibiotic therapy and bacteremia, baseline renal function was not correlated with clinical and microbiological successes in MRSA pneumonia at either end of treatment or at end of study for both treatment groups. No positive association was identified between vancomycin exposures and efficacy in these patients. Higher vancomycin exposures were correlated with an increased risk of nephrotoxicity (e.g., hazards ratio [95% confidence interval] for a 5 μg/ml increase in trough concentration: 1.42 [1.10, 1.82]).

**Conclusions:**

In non-dialysis patients, baseline renal function did not impact the differences in efficacy or nephrotoxicity with treatment of linezolid versus vancomycin in MRSA pneumonia.

**Electronic supplementary material:**

The online version of this article (doi:10.1186/s12882-017-0581-y) contains supplementary material, which is available to authorized users.

## Background

Methicillin-resistant *Staphylococcus aureus* (MRSA) is a common cause of nosocomial pneumonia, a potentially serious infection of hospitalized patients, and renal impairment is a common comorbidity among hospitalized patients with serious infections [1]. Linezolid and vancomycin are both utilized as standard of care for the treatment of nosocomial pneumonia caused by MRSA. In a randomized, double-blind, controlled, multicenter clinical trial, conducted between 2004 and 2010, of linezolid versus (vs.) vancomycin for the treatment of culture-proven MRSA nosocomial pneumonia, the primary efficacy analysis demonstrated treatment difference in favor of linezolid in the MRSA pneumonia patients [2]. Conversely, nephrotoxicity occurred more frequently in the vancomycin group (18.2%; linezolid, 8.4%) [2].

The pharmacokinetics (PK) of linezolid and vancomycin behave differently in subjects with renal impairment. Plasma concentrations of linezolid are not affected in patients with advanced renal impairment [3], while renal dysfunction impairs excretion of vancomycin resulting in a higher systemic exposure with the same dosing regimen [4]. To avoid toxicity, the vancomycin dose in patients with renal impairment must be reduced or the dosing frequency prolonged in order to maintain the exposure comparable to that in patients with normal renal function [4]. Thus, it may be postulated that differences in outcomes existing in MRSA pneumonia patients treated with linezolid and vancomycin may be driven by differences in renal function since it may affect vancomycin exposure. The activity of vancomycin against staphylococcal species has been proposed to be best predicted by area under the concentration curve over a 24-h interval to minimum inhibitory concentration (AUC_0–24_/MIC), and not by the time above MIC. However, the temporal relationship between elevated vancomycin trough concentrations and development of nephrotoxicity can be problematic in patients with unstable renal function. Furthermore, controversy exists regarding the relationship between vancomycin PK-pharmacodynamic (PD) target attainment and clinical outcomes in the treatment of pneumonia. It has been proposed that a vancomycin AUC_0–24_/MIC of at least 400 should be reached in order to maximize the probability of positive clinical outcomes [5–7]. In the face of “MIC creep” in MRSA isolates, a shift of C_min_ from the traditional target of 5–15 μg/ml to a higher range of 15–20 μg/ml has been proposed in order to achieve vancomycin exposure in sufficient excess of the minimum concentration required to inhibit the organism [1, 7]. However, a retrospective analysis found no evidence that higher vancomycin trough concentrations (e.g., ≥15 μg/ml) or AUC_0–24_ values (e.g., ≥400 μg·h/ml) correlated with improved hospital mortality [8]. The authors concluded that aggressive dosing strategies for vancomycin (e.g., trough concentrations exceeding 15 μg/ml) may not offer any advantage over traditional targets of 5 to 15 μg/ml [8–12].

In addition, there are data to suggest that patients with renal insufficiency may be at greater risk for clinical failure from MRSA infections [13]. Hence, to evaluate if baseline renal function has an impact on the efficacy and toxicity of linezolid and vancomycin in the treatment of nosocomial pneumonia caused by MRSA, we performed a retrospective cohort analysis of data from the above mentioned randomized, double-blind, controlled clinical trial [2].

Our primary objectives were to compare efficacy outcomes and nephrotoxicity between linezolid-treated and vancomycin-treated patients with culture-proven MRSA pneumonia by baseline renal function strata (normal, mild/moderate impairment and severe impairment). Additionally, we examined the relationships between vancomycin exposure and PK/PD variables (C_min,_ AUC_0–24_ and AUC_0–24_/MIC) and outcomes (efficacy and nephrotoxicity) for the subset of patients with available vancomycin concentrations and MRSA MIC data.

## Methods

### Study population

The subjects for this retrospective analysis were participants in a prospective, randomized clinical trial that included hospitalized patients ≥18 years old with documented hospital-acquired or healthcare-associated pneumonia, and a baseline respiratory or sputum specimen positive for MRSA.

Data from the modified intent-to-treat (mITT) population, defined as those patients who received at least 1 dose of study drug and had a culture confirmed for MRSA, were used for the retrospective analysis. To avoid any potential bias on comparison between the two treatment groups, mITT patients who had a history of dialysis prior to the study treatment or received dialysis during the study were excluded from this analysis.

### Study design of the prospective clinical trial

A detailed report of this trial is published elsewhere [2]. Provided herein is an abbreviated version of the study design relevant to the current retrospective analysis.

Patients were randomized to receive treatment with intravenous linezolid (600 mg every 12 h [q12h]) or vancomycin (15 mg/kg q12h) for 7–14 days (or 21 days if bacteremia was present). Linezolid and vancomycin solutions were infused over a 60–120 min period. Patients also received an antibiotic for gram-negative coverage without MRSA activity that was discontinued if no gram-negative pathogens were identified.

Vancomycin dose was determined based on the patient’s glomerular filtration rate and adjusted according to serum trough concentrations as per standard of clinical practice at the discretion of an unblinded pharmacist. Trough samples for vancomycin measurement were collected within 30 min to 1 h prior to next scheduled dose on study days 3 and 6, respectively. More samples were also allowed to be collected at different time points at investigator’s discretion. Vancomycin plasma concentrations were determined at the local laboratories per local practice. Final MRSA identification and MIC testing were performed at a central laboratory via broth microdilution methods according to the current Clinical and Laboratory Standards Institute guidelines at the time of study initiation [14].

Clinical and microbiological outcomes were assessed within 5 days of the end of treatment (EOT) and at the end of study (EOS; 7 to 30 days after EOT). Clinical response could be cure, improvement (for EOT visit only), failure or indeterminate, each of which was prospectively defined [2]. For instance, clinical cure was defined as (i) resolution of clinical signs and symptoms of pneumonia compared with baseline, (ii) improvement or lack of progression in chest imaging, and (iii) no requirement for additional antibacterial treatment; clinical improvement was defined as (i) improvement in two or more clinical signs and symptoms of pneumonia compared with baseline, (ii) and (iii) are the same as for clinical cure. Clinical success included clinical cure and improvement. Microbiological response could be documented eradication, presumed eradication, documented persistence, presumed persistence, superinfection, colonization or indeterminate, each of which was prospectively defined [2]. For instance, documented eradication was defined as absence of MRSA from the infection site, and presumed eradication was defined as clinical cure without available microbiological culture data. Microbiological success included the documented eradication and presumed eradication.

Adverse events (AEs) were monitored throughout the study (until 28 days post treatment), and overall survival was assessed at 60 days after therapy.

### Baseline renal function assessment

The traditional Cockcroft-Gault formula [15] was used to calculate the estimated glomerular filtration rate, expressed as creatinine clearance (CLcr, equation below). Patients were categorized into one of three strata according to baseline renal function as follows: CLcr >80 ml/min (normal), CLcr ≥30 to ≤80 ml/min (mild/moderate impairment), and CLcr <30 ml/min (severe impairment).

$$ \mathrm{CCr}=\left\{\left(\left(\mathrm{l}\kern0.5em 40-\mathrm{age}\right)\times \mathrm{weight}\Big)/\Big(72\times \mathrm{SCr}\right)\right\}\times 0.85\kern0.5em \left(\mathrm{if}\kern0.5em \mathrm{female}\right) $$

Note: the correction factor for female subject is 0.85.

### Efficacy assessment

Four efficacy endpoints were included in this analysis: clinical response at EOT, clinical response at EOS, microbiological response at EOT and microbiological response at EOS. Patients with missing data on outcomes were excluded from the analysis.

### Nephrotoxicity assessment

The diagnostic criteria for acute kidney injury (AKI) in this analysis were as follows: an abrupt (within 48 h) reduction in kidney function defined as an absolute increase in serum creatinine of ≥0.3 mg/dl (≥26.4 umol/l) or a 50% increase in serum creatinine (1.5-fold from baseline) if abnormal at baseline [16]. The mITT patients with renal function data missing were excluded from the analysis.

### Vancomycin PK and exposure-response analysis

Multiple population PK models have been developed to describe vancomycin PK [17]. A previously developed PK model for critically ill patients by Llopis-Salvia et al. [18] was adopted here. Since only sparse samples were collected in our study, the PK parameter estimates from the previous model were used as a priori for our model development. Modification was made to the previously developed model as appropriate. Other previously developed model structures were evaluated to ensure the most appropriate model was selected in our analysis [17].

Concentration data were analyzed using a nonlinear mixed-effects population analysis approach with NONMEM version 7.2 (Ellicott City, MD). The first-order conditional estimation (FOCE) method was employed. The graphic processing of the NONMEM output and descriptive summary statistics of PK parameters were performed with R (version 2.12.2). Model selection was based on standard goodness-of-fit criteria including diagnostic plots, precision of parameter estimates and the objective function value. Note that patients who had a history of dialysis prior to the study treatment or received dialysis during the study were also excluded from the PK analysis.

The final PK model was used to generate the posterior Bayesian estimates of individual clearance. Since vancomycin dose and dosing frequency varied in many patients (based on their renal function and C_min_), the vancomycin dose, estimated C_min_ and AUC_0–24_ (calculated as dose/clearance), in each individual referred to the average values during the treatment period. Individual average AUC_0–24_ values were subsequently used for the calculation of the PK-PD index, AUC_0–24_/MIC.

The relationships between vancomycin exposures and PK-PD index (C_min,_ AUC_0–24_ and AUC_0–24_/MIC) and efficacy or nephrotoxicity were explored in mITT patients with these parameters available.

### Statistical analysis

Statistical analyses were performed using the SAS version 9.2 (Cary, NC) software. Statistical comparisons between treatment groups were performed using the Chi-square test or Fisher Exact test for categorical variables and a one-way analysis of variance for continuous variables with significance at a *P* value of <0.05. For each stratified analysis of the efficacy and safety endpoints, the risk difference (linezolid minus vancomycin) and its associated 95% confidence interval (CI) were calculated. The 95% CIs for the risk differences were based on asymptotic normal approximation.

Multivariate logistic regression analyses of patient demographics and clinical characteristics, including treatment (linezolid/vancomycin), were performed to identify variables associated with efficacy outcomes. Covariates that were considered in the multivariate analysis included renal function (3 categories), age, weight, gender, race, Acute Physiology and Chronic Health Evaluation (Apache) II score, pleural effusion (yes/no), bacteremia (yes/no), ventilated at baseline (yes/no), admitted to intensive care unit at baseline (yes/no), pathogen type (MRSA only/MRSA mixed), chest X-ray (bilateral/unilateral), comorbidities at baseline (cardiac, diabetes, neoplastic, renal/urinary, pulmonary, hepatobiliary, gastrointestinal) (yes/no), and prior medications at baseline (antibiotics used to treat anaerobic infections, vasopressors and corticosteroids) (yes/no). Four clinically relevant potential interactions with renal function were selected for evaluation: treatment, age, Apache II score and ventilated at baseline. Covariates that passed the covariate reduction techniques were included in the multivariate logistic regression analysis. A backward elimination approach was used to fit the most parsimonious model with a *P* value of ≤0.05 for staying in the final model. To minimize the risk of over-fitting the model, a bootstrap method was used to assess the frequencies of inclusion of each covariate in the backward elimination process. Covariates with a low rate of inclusion (i.e., <50% of 1000 bootstrap samples) were not included in the final model.

Univariate logistic regression was performed to assess the relationship between vancomycin exposures and PK-PD index (C_min_, AUC_0–24_ and AUC_0–24_/MIC) and efficacy outcomes with or without interaction with baseline renal function. Cox proportional hazards regression was performed to assess the relationship between vancomycin exposures (C_min_ and AUC_0–24_) and AKI with or without interaction with baseline renal function.

## Results

### Patient characteristics

Out of the 448 mITT MRSA patients, 405 non-dialysis patients were identified with a baseline CLcr value available. Among them, 228 (linezolid, *n* = 127 and vancomycin, *n* = 101) had normal renal function (CLcr: >80 ml/min) at baseline, 152 (linezolid, *n* = 73 and vancomycin, *n* = 79) had mild/moderate renal impairment (CLcr: 30–80 ml/min) and 25 (linezolid, *n* = 9 and vancomycin, *n* = 16) had severe renal impairment (CLcr: <30 ml/min).

As shown in Table 1, no significant differences were found in patient demographics and clinical characteristics between the treatment groups within each renal function stratum except for one variable. A difference in race was noted in the severe renal impairment stratum (all the 9 patients in linezolid group were White). The baseline renal function varied inversely with age, which is not unexpected since it is known that the renal function tends to decrease in older adults. Additionally, vancomycin MIC of 1 μg/ml was most prevalent for the MRSA pathogen across strata. The average treatment duration across groups was approximately 10 days.

**Table 1:** Clinical characteristics of mITT patients (*N* = 405) by baseline renal function

	Severe impairment (<30 ml/min)		*P* value*	Mild/moderate impairment (30–80 ml/min)		*P* value*	Normal renal (>80 ml/min)		*P* value*
	LZD (*N* = 9)	VAN (*N* = 16)		LZD (*N* = 73)	VAN (*N* = 79)		LZD (*N* = 127)	VAN (*N* = 101)	
Age (yr); mean (SD)	77 (10)	76 (14)	0.90	72 (13)	72 (10)	0.98	55 (18)	51 (17)	0.11
Male gender; n (%)	3 (33)	7 (44)	0.69	44 (60)	51 (65)	0.62	90 (71)	68 (67)	0.57
Weight (kg); mean (SD)	64 (12)	72 (24)	0.40	72 (18)	71 (18)	0.87	83 (25)	83 (22)	0.94
Race; n (%)									
White	9 (100)	8 (50)	0.04	52 (71)	50 (63)	0.27	83 (65)	76 (75)	0.08
Black	0	3 (19)		6 (8)	11 (14)		18 (14)	15 (15)	
Asian	0	5 (31)		10 (14)	16 (20)		20 (16)	5 (5)	
Other	0	0		5 (7)	2 (3)		6 (5)	5 (5)	
CLcr; mean (SD)	22 (6)	22 (6)	0.98	58 (14)	59 (14)	0.51	139 (56)	154 (66)	0.08
Apache II score; n/mean (SD)	9/21 (7)	16/20 (7)	0.80	71/19 (6)	75/18 (6)	0.60	125/16 (6)	101/16 (5)	0.98
Bacteremia; n (%)	0	2 (13)	0.52	15 (21)	17 (22)	1.0	10 (8)	13 (13)	0.27
Ventilated at baseline; n (%)	7 (78)	11 (69)	1.0	45 (62)	51 (65)	0.74	91 (72)	77 (76)	0.45
ICU at baseline; n (%)	7 (78)	13 (81)	1.0	59 (81)	67 (85)	0.53	106 (84)	90 (89)	0.25
MIC (μg/ml); n (%)									
0.5	2 (22)	0	0.05	3 (4)	2 (3)	0.73	11(9)	12 (12)	0.33
1	5 (56)	15 (94)		55 (75)	59 (75)		98 (77)	69 (68)	
≥ 2	2 (22)	1 (6)		5 (7)	9 (11)		5 (4)	9 (9)	
unknown	0	0		10 (14)	9 (11)		13 (10)	11 (11)	
Treatment duration (days); mean (SD)	11 (5)	10 (5)	0.60	9 (4)	10 (4)	0.69	10 (4)	10 (4)	0.57

*mITT* modified intent to treat, *LZD* linezolid, *VAN* vancomycin, *SD* standard deviation, *n* number of subjects, *CLcr* creatinine clearance, *Apache II score* Acute Physiology and Chronic Health Evaluation II score, *ICU* intensive care unit, *MIC* minimum inhibitory concentration

*for continuous variables, one-way analysis of variance was used; for categorical variables, the Chi-square test or Fisher Exact test, as appropriate, were used

### Efficacy analysis

The differences in clinical and microbiological successes between treatment groups (linezolid minus vancomycin) by renal function strata are presented in Table 2, in comparison with the overall differences in the mITT patients. Except for clinical success at EOS, the differences remained significant for the other 3 endpoints in patients with normal renal function. For patients with renal impairment, all the endpoints had comparable responses in both treatment groups, with one exception (microbiological success at EOT).

**Table 2:** Differences in clinical and microbiological successes and nephrotoxicity by treatment and baseline renal function in mITT patients

Parameter	Baseline renal function strata^a,b^	Linezolid n/N (%)^c^	Vancomycin n/N (%)^c^	Absolute risk differences in success/toxicity rates % (95% CI)^c^
Clinical Success at EOT	All mITT patients	161/201 (80)	145/214 (68)	12.3 (4.0, 20.7)*
	Normal	99/117 (85)	65/95 (68)	16.2 (4.8, 27.6)*
	Mild/moderate impairment	48/63 (76)	53/77 (69)	7.4 (−7.4, 22.1)
	Severe impairment	5/7 (71)	10/15 (67)	4.8 (−36.3, 45.9)
Clinical Success at EOS	All mITT patients	102/186 (55)	92/205 (45)	10.0 (0.1, 19.8)*
	Normal	65/108 (60)	44/92 (48)	12.4 (−1.4, 26.1)
	Mild/moderate impairment	29/59 (49)	32/73 (44)	5.3 (−11.8, 22.4)
	Severe impairment	4/8 (50)	5/13 (38)	11.5 (−32.0, 55.1)
Microbiological Success at EOT	All mITT patients	161/203 (79)	127/218 (58)	21.1 (12.5, 29.7)*
	Normal	93/117 (80)	56/96 (58)	21.2 (8.9, 33.4)*
	Mild/moderate impairment	50/65 (77)	44/79 (56)	21.2 (6.2, 36.2)*
	Severe impairment	5/7 (71)	11/15 (73)	−1.9 (−42.2, 38.4)
Microbiological Success at EOS	All mITT patients	111/195 (57)	96/209 (46)	11.0 (1.3, 20.7)*
	Normal	67/111 (60)	44/95 (46)	14.0 (0.5, 27.6)*
	Mild/moderate impairment	33/65 (51)	36/74 (49)	2.1 (−14.5, 18.8)
	Severe impairment	5/8 (63)	5/13 (38)	24.0 (−18.7, 66.8)
Occurrence of acute kidney injury (AKI)	All mITT patients	18/214 (8)	39/214 (18)	−9.8 (−16.2, −3.4)*
	Normal	14/126 (11)	21/99 (21)	−10.1 (−19.8, −0.4)*
	Mild/moderate impairment	5/71 (7)	13/78 (17)	−9.6 (−19.8, 0.6)
	Severe impairment	0/8 (0)	3/15 (20)	−20.0 (−40.2, 0.2)

*EOT* end of treatment, *EOS* end of study, *CI* confidence interval

*P-value <0.05, which was based on Chi-square test or Fisher Exact test

^a^Estimated creatinine clearance for renal function was assessed as a categorical variable: normal = >80 ml/min, mild/moderate impairment = 30–80 ml/min, and severe impairment = <30 ml/min

^b^There were 228 patients with normal renal function (linezolid, *n* = 127; vancomycin, *n* = 101), 152 patients with mild/moderate renal impairment (linezolid, *n* = 73; vancomycin, *n* = 79), and 25 patients with severe renal impairment (linezolid, *n* = 9; vancomycin, *n* = 16)

^c^Percentages were calculated excluding missing data in this analysis

The multivariate analysis of each of the 4 efficacy endpoints demonstrated that the baseline renal function was not an independent predictor of clinical and microbiological successes in the MRSA pneumonia patients (all *P* values >0.05), when controlling for other variables in the model (Table 3). Baseline renal function also did not show significance in any pairwise comparisons. In this analysis, treatment remained as an independent predictor of all efficacy endpoints except for clinical success at EOS (it was forced into the final model to illustrate the treatment effect) (Table 3). In addition, renal function did not modify the relationships between the 4 variables of interest (treatment, age, Apache II score, and ventilated at baseline) and each efficacy endpoint (data on file).

**Table 3:** Multivariate analysis: predictors of clinical and microbiological successes in mITT patients (*N* = 448)^a^

Parameter	Variable	*P* value	Odds ratio (95% CI)
Clinical Success at EOT	Gastrointestinal comorbidity (yes vs. no)	0.069	0.6 (0.4, 1.0)
	Vasopressors at baseline (yes vs. no)	0.005	0.4 (0.2, 0.8)
	Peripheral vascular disease at baseline (yes vs. no)	0.021	3.0 (1.2, 7.8)
	Treatment (linezolid vs vancomycin)	0.014	1.9 (1.1, 3.1)
	Bacteremia (yes vs. no)	0.008	0.4 (0.2, 0.8)
	Renal function (CLcr, ml/min)^b^	Overall: 0.775	
	30–80 vs. >80	0.609	0.9 (0.5, 1.5)
	<30 vs. >80	0.548	0.7 (0.3, 2.1)
	<30 vs. 30–80	0.735	0.8 (0.3, 2.4)
Clinical Success at EOS	Cardiac comorbidity (yes vs. no)	0.037	0.6 (0.4, 1.0)
	Vasopressors at baseline (yes vs. no)	0.004	0.4 (0.2, 0.8)
	Treatment (linezolid vs vancomycin)^c^	0.096	1.4 (0.9, 2.2)
	Bacteremia (yes vs. no)	0.074	0.5 (0.3, 1.1)
	Pathogen type (MRSA mixed vs. MRSA only)	0.034	0.6 (0.4, 1.0)
	Renal function (CLcr, ml/min)^b^	Overall: 0.673	
	30–80 vs. >80	0.506	0.9 (0.5, 1.4)
	<30 vs. >80	0.477	0.7 (0.3, 1.8)
	<30 vs. 30–80	0.703	0.8 (0.3, 2.2)
Microbiological Success at EOT	Vasopressors at baseline (yes vs. no)	0.018	0.5 (0.3, 0.9)
	Treatment (linezolid vs vancomycin)	<0.001	2.6 (1.7, 4.2)
	Hepatobiliary comorbidity (yes vs. no)	0.054	0.5 (0.3, 1.0)
	Renal function (CLcr, ml/min)^b^	Overall: 0.550	
	30–80 vs. >80	0.476	0.8 (0.5, 1.4)
	<30 vs. >80	0.513	1.4 (0.5, 4.0)
	<30 vs. 30–80	0.329	1.7 (0.6, 4.7)
Microbiological Success at EOS	Treatment (linezolid vs vancomycin)	0.040	1.6 (1.0, 2.4)
	Chest X-ray (Bilateral vs Unilateral)	0.005	0.5 (0.3, 0.8)
	Pleural effusion (yes vs. no)	0.021	1.7 (1.1, 2.7)
	Renal function (CLcr, ml/min)^b^	Overall: 0.691	
	30–80 vs. >80	0.422	0.8 (0.5, 1.3)
	<30 vs. >80	0.643	0.8 (0.3, 2.0)
	<30 vs. 30–80	0.937	1.0 (0.4, 2.5)

*EOT* end of treatment, *EOS* end of study, *CLcr* creatinine clearance, *CI* confidence interval

^a^Missing data were excluded in this model

^b^Renal function was forced into the model

^c^Treatment was forced into the model

### Nephrotoxicity analysis

Similar to efficacy endpoints, the difference remained significant for the AKI occurrence in patients with normal renal function (lower AKI incidence in linezolid treatment group) (as shown in Table 2). In the vancomycin treatment group, the AKI occurrence was similar across 3 renal function strata; in the linezolid treatment group, patients with normal renal function appeared to have more AKI reported than those with renal impairment, indicating no correlation between AKI occurrence in these MRSA pneumonia patients and their baseline renal function impairment regardless of the treatment.

### Vancomycin population PK analysis

Vancomycin plasma concentration data from 304 non-dialysis patients (456 observations) were used for the analysis. The final PK model was a 2-compartment model with first-order elimination. Inter-subject variability in the PK parameters was modeled using multiplicative exponential random effects, and residual error (within-subject variability) was modeled with a proportional error. This PK model described the concentration data well and the diagnostic plots are presented in Additional file 1: Figure S1. The forms of the equation for the model parameters are presented below, and the parameter estimates from the final model are presented in Table 4.

$$ \begin{array}{l} CL={\theta}_{CL}\cdot \left( CLcr/80\right)\\ {}{V}_1={\theta}_{V_1}\cdot \left( WT/70\right)\\ {}{V}_2={\theta}_{V_2}\cdot \left( WT/70\right)\\ {} Q={\theta}_Q\end{array} $$

**Table 4:** Vancomycin parameter estimates from the population PK model

Parameter	Typical Value (%RSE^a^)	Inter-individual Variability %CV (%RSE^a^)
CL (L/h/80 ml/min), θ_CL_	3.12 (6.1)	47.5 (13.2)
V_1_ (L/70 kg), θ_V1_	43.5 (22.8)	92.2 (40.4)
Q (L/h), θ_Q_	8.66 (51.6)	NE
V_2_ (L/70 kg), θ_V2_	45.8 (19.1)	81.5 (30.1)
Residual Error Parameter		
σ^2^_1prop_ (%)	23.2 (15.6)	

*CL*clearance, *V*_*1*_central volume of distribution, *Q* inter-compartmental clearance, *V*_*2*_peripheral volume of distribution, *σ* ^*2*^_*1prop*_ proportional component of the residual error model, *NE* not estimated

^a^%RSE: percent relative standard error of the estimate = SE/parameter estimate * 100 (for variability terms this is the %RSE of the variance estimate)

CL = Clearance, CLcr = creatinine clearance, V_1_ = central volume of distribution, WT = baseline body weight, V_2_ = peripheral volume of distribution, Q = inter-compartmental clearance, θ = estimate of fixed effect in NONMEM.

There were 133 mITT patients with estimated vancomycin exposures and PK-PD index (C_min_, AUC_0–24_ and AUC_0–24_/MIC) available. Their corresponding vancomycin dose, clearance, exposure parameters and PK-PD index are summarized by baseline renal function strata in Table 5. As expected, vancomycin daily dose and clearance in patients with renal impairment were lower than those in patients with normal renal function. Although average vancomycin exposures (C_min_ and AUC_0–24_) in patients with renal impairment were slightly higher than those in patients with normal renal function, there were substantial overlaps in exposure distributions among them. It indicated that vancomycin exposures in patients with renal impairment were comparable to those in patients with normal renal function. Similarly, average AUC_0–24_/MIC values in patients with renal impairment were slightly higher than that in patients with normal renal function, and there was a substantial overlap in AUC_0–24_/MIC distributions among them.

**Table 5:** Summary of vancomycin dose, estimated PK and PK-PD index parameters by baseline renal function

Parameters^a^		Normal renal (>80 ml/min) (*n* = 67)	Mild/moderate impairment (30–80 ml/min) (*n* = 53)	Severe impairment (<30 ml/min) (*n* = 13)
Daily dose (mg/kg/day)	Mean (CV%)	29.4 (36)	23.2 (39)	13.5 (58)
	Median (range)	28.6 (9.3–60.4)	23.5 (8.2–53.0)	11.6 (4.0–35.3)
Clearance (CL, l/h)	Mean (CV%)	5.7 (44)	2.7 (38)	1.0 (47)
	Median (range)	5.3 (0.8–11.3)	2.8 (0.9–4.9)	0.9 (0.3–2.0)
AUC_0–24_ (μg·h/ml)	Mean (CV%)	428 (38)	524 (33)	548 (22)
	Median (range)	404 (153–1121)	484 (226–1203)	516 (384–777)
AUC_0–24_/MIC	Mean (CV%)	458 (58)	490 (41)	524 (26)
	Median (range)	394 (76–1320)	472 (108–1203)	514 (309–777)
Trough concentration (C_min_, μg/ml)	Mean (CV%)	11.9 (54)	16.0 (40)	18.4 (25)
	Median (range)	10.6 (3.2–38.6)	15.0 (6.7–42.5)	18.0 (12.4–28.7)

*CV%* coefficient variation in percentage, *AUC*_*0–24*_ area under the curve over 24 h, *MIC* minimum inhibitory concentration

^a^The average values during the treatment period in each individual were used for summary statistics

### Vancomycin exposure-response analysis

The univariate logistic regression analysis showed that none of the exposure/PK-PD variables (C_min_, AUC_0–24_ and AUC_0–24_/MIC) were positively associated with any of the efficacy endpoints (clinical and microbiological successes at EOT and EOS, respectively). Baseline renal function did not modify the relationships between vancomycin exposure/PK-PD variables and efficacy outcomes.

Based on the Cox proportional hazards regression analysis, a statistically significant association was identified between vancomycin exposures (C_min_ and AUC_0–24_) and the risk of AKI occurrence. Specifically, for a 5 μg/ml increase in vancomycin trough concentration (C_min_), patients have 1.42 (95% CI: 1.10, 1.82) times higher risk of developing AKI (*P* = 0.007). Similarly, for a 50 μg·h/ml increase in vancomycin total exposure (AUC_0–24_), patients have 1.15 (95% CI: 1.04, 1.27) times higher risk of developing AKI (*P* = 0.006). Baseline renal function did not modify the relationships between vancomycin exposures and AKI occurrence.

## Discussion

We conducted this retrospective analysis of a subpopulation of patients from a prospective, randomized, controlled clinical trial to determine whether baseline renal function impacted treatment efficacy and nephrotoxicity of linezolid and vancomycin in MRSA pneumonia patients. Evidence assessing the effectiveness of antimicrobials other than vancomycin for the treatment of serious infections caused by MRSA isolates in patients with renal impairment is limited [19–21]. Only a few review studies, all with methodological limitations, attempted to address this question.

Our data showed that baseline renal function is not a predictor of efficacy in MRSA pneumonia patients with other variables (eg, baseline comorbidities, etc.) adjusted for the model in the multivariate analysis. It indicated that the efficacy of linezolid and vancomycin (with an optimized dosing regimen) for the treatment of MRSA pneumonia was not affected by the renal function impairment. In addition, there was no correlation between the occurrence of AKI in the MRSA pneumonia patients and their baseline renal function impairment status in this study. It suggested that the renal function impairment at baseline is not a risk factor for the occurrence of nephrotoxicity in MRSA pneumonia patients receiving vancomycin or linezolid treatment.

The stratified analysis of efficacy and nephrotoxicity based on renal function strata had consistent results as the primary analysis using all mITT patients (shown in Table 2) [2]. The treatment difference in favor of linezolid in MSRA pneumonia patients still exists although statistical significance was not demonstrated in all comparison groups due to relatively small sample size in patients with renal impairment.

Since only sparse vancomycin PK samples were collected in this study, vancomycin concentration data from all 305 non-dialysis patients were used to increase the robustness of the data in order to facilitate the PK model development. For subsequent exposure-response analysis, only evaluable mITT patients (*n* = 133) were included for analysis.

As shown in Table 5, vancomycin trough concentrations (C_min_) in patients with renal impairment were comparable to those in patients with normal renal function with slightly higher mean values (normal, mild/moderate, severe: 11.9, 16.0, 18.4 μg/ml). Similarly, vancomycin total exposures (AUC_0–24_) in patients with renal impairment were comparable to those in patients with normal renal function with slightly higher mean values (normal, mild/moderate, severe: 428, 524, 548 μg·h/ml). This confirmed that vancomycin doses were adjusted appropriately in patients with renal impairment in this study.

The lack of positive association between vancomycin exposure/PK-PD variables (C_min_, AUC_0–24_ and AUC_0–24_/MIC) and efficacy endpoints was observed in this analysis, and baseline renal function also did not modify these relationships. This indicated that maintaining vancomycin C_min_ over 15 to 20 μg/ml or AUC_0–24_ over 400 μg·h/ml in order to achieve successful outcomes needs further validation. On the other hand, higher vancomycin exposures were correlated with an increased risk of AKI occurrence, which is consistent with the findings from a previous retrospective analysis [22] and a more recent prospective study [23]. Therefore, it may not be necessary to push vancomycin exposure too high in order to achieve successful outcomes since minimizing the risk of nephrotoxicity should also be taken into consideration during the management of MRSA pneumonia patients.

There are a few limitations with this analysis. First, due to the nature of this type of retrospective analysis, it may introduce selection bias. Fortunately, there were no significant differences found in patient demographics and clinical characteristics between the treatment groups within each renal function stratum except for one variable (race) in our analysis. Second, there were a limited number of patients with renal impairment, especially those with severe renal impairment, and there were an imbalanced number of patients between treatment groups in the severe renal impairment stratum. Third, patients receiving dialysis during the study or with a history of dialysis were excluded from the analysis because of different volume of distribution; hence, the results from this analysis cannot be generalized to patients on dialysis. Fourth, the height was not collected in this study and creatinine clearance was calculated using the actual body weight instead of ideal body weight, which may have an impact on the assignment of the renal function strata. Fifth, only vancomycin trough concentrations were available and used together with parameter estimates from a previous model (as a priori) for the PK model development, which may introduce potential bias on the estimation of individual clearance, in turn affecting the estimation of AUC_0–24_. Finally, the diagnosis of MRSA pneumonia in this study was based on the cultures from either respiratory or sputum specimens instead of quantitative bronchoalveolar lavage cultures only, which was acceptable at the time of study conduct. It is thought using cultures from both respiratory and sputum specimens for diagnosis may lead to over diagnosis. Nonetheless, our analysis used the data from one single study, which minimized the heterogeneity of the data. In addition, patients in our study were enrolled from different geographical regions (eg, North America, South America, Europe, Africa and Asia), making our results more representative.

## Conclusions

In summary, for this subgroup of patients with confirmed MRSA pneumonia, baseline renal function did not contribute to differences in efficacy and safety with treatment of linezolid vs. vancomycin. No positive association was found between vancomycin exposure/PK-PD variables (C_min_, AUC_0–24_ and AUC_0–24_/MIC) and efficacy endpoints, and baseline renal function did not modify these relationships. Higher vancomycin exposures were correlated with an increased risk of AKI occurrence.

## Additional file

Additional file 1: Figure S1. Vancomycin basic goodness of fits. (DOC 53 kb)

## Abbreviations

APACHE: Acute Physiology and Chronic Health Evaluation
AKI: Acute kidney injury
AUC: Area under the curve
CI: Confidence interval
EOS: End of study
EOT: End of treatment
mITT: modified intent-to-treat
MRSA: Methicillin resistant *Staphylococcus aureus*
