# Supplementary material for: Clinical outcomes of linezolid and vancomycin in patients with nosocomial pneumonia caused by methicillin-resistant Staphylococcus aureus stratified by baseline renal function: a retrospective, cohort analysis
Source: BMC Nephrol. 2017 May 22;18:168. doi: 10.1186/s12882-017-0581-y (PMC5440938; doi:10.1186/s12882-017-0581-y)
Supplement: Additional file 1: Figure S1. — Vancomycin basic goodness of fits. (DOC 53 kb) [file 12882_2017_581_MOESM1_ESM.doc]

Figure S1. Vancomycin Basic Goodness of Fit Plots

Key – open symbols are observed data, dashed line is the line of unity or line of identity, solid line is the loess smooth.
